# Differences in Anthocyanin Accumulation Patterns and Related Gene Expression in Two Varieties of Red Pear

**DOI:** 10.3390/plants10040626

**Published:** 2021-03-25

**Authors:** Jianlong Liu, Zhiwei Deng, Hongwei Sun, Jiankun Song, Dingli Li, Shaoling Zhang, Ran Wang

**Affiliations:** 1 Lab of Pear Genetic Improvement and Germplasm Innovation, Qingdao Agricultural University, Qingdao 266109, China; 201901068@qau.edu.cn (J.L.); zhiweideng@stu.qau.edu.cn (Z.D.); shw@stu.qau.edu.cn (H.S.); 200601048@qau.edu.cn (J.S.); lidingli@qau.edu.cn (D.L.); 2 Centre of Pear Engineering Technology Research, Nanjing Agricultural University, Nanjing 210095, China

**Keywords:** anthocyanin, coloration, light signal, calcium, hormone

## Abstract

Red pear is a popular fruit that is appreciated for its attractive and distinctive appearance and mild flavor. In this study, we investigated the mechanism underlying the red coloration of pear skin using the ‘Xinqihong’ cultivar—which was selected as a spontaneous bud sport mutant of the ‘Xinli 7′(*Pyrus betulifolia* Rehd.) variety and has a stronger red color that is retained in the mature fruit—as an experimental model. We compared the phenotype and gene expression patterns of the two varieties and found no significant differences at the early stage of fruit development. However, although the red color of ‘Xinli 7′ fruits began to fade 107 days after full bloom, that of ‘Xinqihong’ fruits persisted until the time of harvest. Transcriptome sequencing identified 639 genes that were differentially expressed between the two varieties, including genes related to light, calcium, and hormone signaling (e.g., *PbPIF3*, *PbGA2ox*, and the calmodulin related genes). Moreover, anthocyanin biosynthesis genes were downregulated as the red color of ‘Xinli 7′ fruits faded. These results provide insight into the molecular basis of color differences in red pears that can be useful for improving its fruit quality and commercial value.

## 1. Introduction

Fruit peel color is a key trait for judging fruit quality. Anthocyanin is a water-soluble flavonoid and natural colorant that accumulates in many plant tissues, including fruit peel [1] and flower petals [2]. As an antioxidant phenolic compound, anthocyanin has anti-inflammatory and anticancer activities, as well as reported health benefits [3].

Asian red-skinned pears develop their characteristic color when they are nearly ripe [4], in contrast to the European red pear that undergoes color change at the beginning of fruit development and loses the red color as it matures. The anthocyanin contents of the ‘Hongyun’ pear and its mutant ‘Yunhongli 1′ and ‘Meirensu’ are maximal in the mature fruit under the natural growing conditions in Yunnan, China [5]. However, the red sand pear variety ‘Mantianhong’ and its mutant ‘Aoguan’ show a fluctuating color, like European pears [6]. Thus, coloring mechanisms differ across pear varieties. Anthocyanins, chlorophyll, and carotenoids are the main pigments responsible for the fruit color in pears [7]. More specifically, the concentration and composition of anthocyanins determine the color of pear peel.

Genes regulating anthocyanin biosynthesis have been cloned in pears [4], including those encoding the enzymes phenylalanine ammonia lyase (*PAL*), chalcone synthase (*CHS*), chalcone isomerase (*CHI*), flavonoid 3-hydroxylase (*F3H*), dihydroflavonol 4-reductase (*DFR*), anthocyanin synthase/leucoanthocyanidin dioxygenase (*ANS*/*LDOX*), and UDP glucose:flavonoid 3-O-glucosyltransferase (*UFGT*) as well as the MYB, basic helix-loop-helix (*bHLH*), and WD40 transcription factors (TFs) [8] that form the MYB—bHLH—WD40 (MBW) transcriptional complex [9]. R2R3-MYB has been identified in various fruit crops [10]. MYB10 and MYB10.1 interact with bHLHs to promote anthocyanin accumulation in pears. In apples and *Arabidopsis*, constitutive photomorphogenic 1 (COP1) regulates fruit color by interacting with MYB1 and the cold-induced bHLH3. Other transcription factors (TFs) participate in anthocyanin biosynthesis through interaction with the MBW complex. Environmental factors—especially light—also regulate anthocyanin biosynthesis [11,12]: under low light or dark conditions, the components of the MBW complex are downregulated, resulting in the decreased expression of structural genes and anthocyanin content.

Plant hormones regulate various physiologic processes, including growth and development [13,14,15]. In apples, treatment with the ethylene inhibitor 1-methylcyclopropene inhibited ethylene release and blocked anthocyanin accumulation [16]. The plant hormone abscisic acid (ABA), an essential regulator of plant growth and development, was shown to promote chlorophyll degradation and anthocyanin biosynthesis in apple pericarp, whereas the ABA synthesis inhibitor, fluoridone, prevented the accumulation of anthocyanin in apple fruits. In addition to ethylene and ABA, gibberellin also stimulates the biosynthesis of anthocyanin [17,18,19].

Calcium signaling regulates the anthocyanin metabolic pathway in plants. In *Arabidopsis*, sucrose-induced sugar absorption was modulated by endogenous calcium levels, and an increased concentration of sugar induced anthocyanin accumulation through the regulation of genes in the anthocyanin biosynthesis pathway [20]. Immersion in CaCl_2_ increased anthocyanin content in jujube fruit, which was associated with increased firmness, ascorbic acid concentration, and shelf life [21].

Coloration patterns differ across pear varieties, although the underlying regulatory mechanisms are not fully understood. To address this issue, in the present study, we compared the phenotype, physiologic properties, and gene expression profiles of the ‘Xinli 7′ (*Pyrus betulifolia* Rehd.) red pear cultivar and its bud spontaneous mutations ‘Xinqihong’, which has fruits with a stronger red color.

## 2. Results

### 2.1. Phenotype of ‘Xinli 7′ and ‘Xinqihong’ Red Pear Varieties

**Figure 1:**
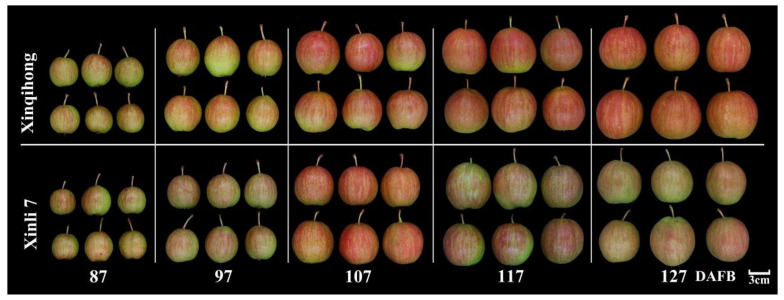
Phenotype of ‘Xinqihong’ and ‘Xinli 7′ red pear varieties at different stages of fruit development. (DAFB: days after full bloom).

‘Xinqihong’ was established as a spontaneous bud sport mutant of ‘Xinli 7′. ‘Xinli 7′ and ‘Xinqihong’ differ in terms of fruit growth, development, and phenotype. ‘Xinqihong’ had a larger area of red color and more intense coloration than ‘Xinli 7′ (Figure 1). Single fruit weight, soluble solid content, and fruit hardness were higher for ‘Xinqihong’ than for ‘Xinli 7′, whereas other characteristics did not differ significantly between the 2 varieties (Table 1).

### 2.2. Anthocyanin Accumulation in ‘Xinli 7′ and ‘Xinqihong’

To determine whether the distinct coloration patterns of ‘Xinqihong’ and ‘Xinli 7′ were due to differences in anthocyanin content, fruits were collected at different developmental stages, i.e., 87, 97, 107, 117, and 127 days after full bloom (DAFB). Gross examination revealed that the skin color of ‘Xinqihong’ fruit gradually intensified, while that of ‘Xinli 7′ faded before the fruit reached maturity. The color parameters (L*, a*, b*, and C*) of the peel were measured and compared (Table 2). The two cultivars showed significant differences in peel color at 107 DAFB, as evidenced by the higher value of a* for ‘Xinqihong’ compared to ‘Xinli 7′ peel.

**Figure 2:**
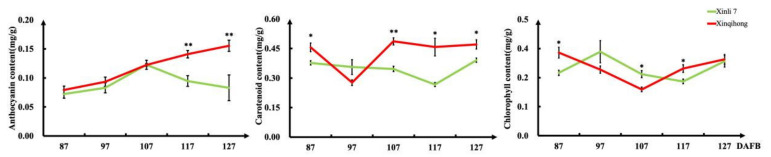
Pigment content of fruit peel at different stages of fruit development. (Student’s *t*-test, * *p* < 0.05; ** *p* < 0.01.

We measured the levels of pigment molecules in the fruit peel at different fruit development stages (Figure 2) and found that the anthocyanin content increased rapidly from 87 DAFB in both red pear varieties. The anthocyanin concentration in ‘Xinli 7′ reached a peak at 107 DAFB before declining, whereas that in ‘Xinqihong’ continued to increase. Carotenoid content in the pericarp was significantly higher in ‘Xinqihong’ than in ‘Xinli 7′ at 107 DAFB. There were no differences in chlorophyll content between the two cultivars at any stage.

### 2.3. Comparative Transcriptome Analysis

We performed RNA sequencing (RNA-seq) to obtain transcriptome profiles of ‘Xinqihong’ and ‘Xinli 7′. High-quality libraries (mapping rate >77% and Q20 and Q30 values >90%) at 87, 107, and 127 DAFB were obtained for the fruit skin of Xinli (Xinli 7_1, Xinli 7_2, and Xinli 7_3, respectively) and Xinqihong (Xinqihong 7_1, Xinqihong 7_2, and Xinqihong 7_3, respectively) (Supplementary Table S6).

**Figure 3:**
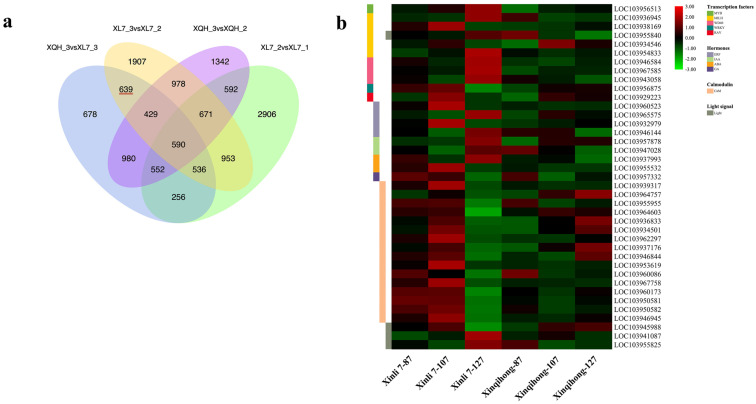
Analysis of DEGs. (**a**) Venn diagram of DEGs at 3 stages of fruit development (87, 107, and 127 DAFB) identified by RNA-seq in 2 red pear cultivars (‘Xinli 7′ and ‘Xinqihong’). (**b**) Fragments per kilobase of transcript per million mapped reads (FPKM) levels of candidate genes encoding TFs, hormones, calmodulin, and light signaling pathway components.

Differentially expressed genes (DEGs) were identified in the comparison of Xinli 7_2 vs. Xinli 7_3 that did not overlap with those identified in the comparison of Xinqihong_2 vs. Xinqihong_3. After removing the DEGs from the Xinli 7_1 vs. Xinli 7_2 and Xinli 7_3 vs. Xinqihong_3 comparisons, 639 DEGs remained (Figure 3a and Supplementary Table S1); these were selected for further analysis as candidate genes related to the fading of the red color of ‘Xinli 7′ fruit peel.

### 2.4. Functional Analysis of DEGs

We identified genes involved in anthocyanin biosynthesis among the DEGs (Figure 3b), including those encoding flavanone-3-hydroxylase (LOC103952304) and anthocyanidin reductase (LOC103957769). In ‘Xinli 7′, the former was significantly upregulated at 127 DAFB. Meanwhile, we analyzed the key structural genes in anthocyanin synthesis by qRT-PCR. Except for *PbPAL*, the expression levels of *PbCHS*, *PbCHI*, *PbDFR*, *PbANS*, *PbUFGT* in the ‘Xinqihong’ pear were significantly higher than that in the ‘Xinli 7′ pear at 107 DAFB (Figure S1).

Nine DEGs were related to the plant hormones ethylene, auxin, ABA, and gibberellin (Figure 3b). Five of the genes were upregulated in ‘Xinli 7′ compared to ‘Xinqihong’ at 87 and 107 DAFB, whereas the gibberellin gene was more highly expressed in ‘Xinqihong’ than in ‘Xinli 7′ at 127 DAFB. These genes may be involved in the color fading phenotype of ‘Xinli 7′.

There were 16 DEGs predicted to encode TFs in seven families (Figure 3b)—namely, *PbbHLH*, *PbMYB*, *PbWD40*, *PbbZIP*, *PbRAV*, *PbWRKY*, and *PbHST*. Six of the genes showed significantly different expression (|log_2_[fold change]| > 1) (Supplementary Table S2) between the two cultivars. Additionally, the expression of *PbWRKY* and *PbERF* changed over the course of fruit development (Figure 3b). Three genes encoding *PbHSTs* and two encoding *PbbZIPs* showed opposite trends during late development in both ‘Xinqihong’ and ‘Xinli 7′.

Two DEGs involved in the light signal transduction pathway and photomorphogenesis were also identified (Figure 3b). These genes were annotated as the light-inducible proteins common plant regulatory factor 2 (CPRF2; LOC103945988) and light-dependent short hypocotyls 10 (LSH10; LOC103941087) (Supplementary Table S3). *PbPIF3* was also identified as a regulatory factor associated with the light signaling pathway.

The synthesis of anthocyanin is regulated by calcium. We identified 16 DEGs that were related to calcium signaling (Figure 3b). The relative expression levels of these 16 genes were higher in ‘Xinli 7′ than in ‘Xinqihong’ at the early stage of fruit development (87 and 107 DAFB). However, the opposite trend was observed at the later stage (127 DAFB). The expression levels of 11 of the genes differed significantly between the two cultivars (|log_2_[fold change] > 1) (Supplementary Table S5).

## 3. Discussion

Pears with a red color are appealing to consumers [22]. Spontaneous bud sport mutants are useful for improving specific traits of superior cultivars. For example, among European pear cultivars, ‘Max Red Bartlett’ was discovered in 1938 as the red-colored spontaneous bud sport mutant of the green ‘Bartlett’ pear in the Yakima Valley of Washington state, United States. Other examples of red pear varieties resulting from spontaneous bud sport mutations include ‘Red Sensation Bartlett’, ‘Rosired Bartlett’, and ‘Bon Rouge’ from ‘Bartlett’; ‘Red D’Anjou’ from ‘D’Anjou’ [23]; ‘Red Clapp’ (Starkrimon) from ‘Clapp’s Favorite’; and ‘Red Nanguo’ from ‘Nanguo’ [4]. ‘Xinli 7′ is a high-quality, early maturing, and storage-resistant red pear variety, but like most red pear varieties has few red areas on the peel. The strongest red color is observed at the middle stage of fruit development as the anthocyanin level in the peel peaks before declining as a result of the dual effects of high temperature-induced degradation during ripening and dilution caused by fruit expansion [24,25]. ‘Xinqihong’ was derived from ‘Xinli 7′ and has a more intense red color at maturity, similar to the red European pear varieties ‘Rosired Bartlett’ and ‘Rogue Red’ [22] that do not fade after the fruit is harvested.

We carried out a comparative analysis of ‘Xinqihong’ and ‘Xinli 7′ in order to identify the molecular basis for their differences in coloration. The anthocyanin content of ‘Xinqihong’ was higher than that of ‘Xinli 7′ at 107 and 127 DAFB, which was in accordance with their color difference parameters. We performed a transcriptomic analysis to identify DEGs that were potentially responsible for the observed difference in peel color between the two cultivars. Anthocyanin biosynthesis involves the regulation of structural genes [26], most of which have been isolated and cloned in pears [4,27]. Only two of these genes were identified by RNA-seq in our study (Supplementary Table S2), and their expression levels did not decrease in the later stages of fruit development in ‘Xinli 7′ and ‘Xinqihong’, suggesting that differences in anthocyanin production are not responsible for the fading of the red color in mature ‘Xinli 7′ fruits. Genes encoding TFs were also differentially expressed between the two red pear varieties (Supplementary Table S2); they were significantly upregulated at the late stage of fruit development in ‘Xinli 7′. MYB10 and its homolog MYB110a [28] are key activators of the anthocyanin biosynthesis pathway in Rosaceae [29]. In this study, *PbMYB10* showed significantly different expression between the two cultivars; the low expression level in ‘Xinli 7′ suggests that its downregulation contributed to color fading in this cultivar.

Genes encoding three WD40 genes as well as bHLHs were identified as DEGs. *PbPIF3* was significantly upregulated in ‘Xinli 7′ compared to ‘Xinqihong’. PIF3 regulates light signaling and anthocyanin biosynthesis in fruit. PIFs and HY5 act antagonistically in many regulatory processes, but PIF3 requires HY5 to bind to the promoter of anthocyanin biosynthesis genes and induce their expression [30,31]. Thus, PbPIF3 may play a role in the fading of color in the ‘Xinli 7′ pear. Three WD40 TFs were also identified. MYB, bHLH, and WD40 TFs are associated with distinct regulatory mechanisms of anthocyanin biosynthesis in occidental vs. oriental pears [4]. The RNA-seq analysis identified four genes encoding light signaling-related genes as DEGs (Supplementary Table S3). A previous study investigating the effects of differences in light quality on fruit color in ‘Hongzaosu’ pears found that red light had little effect on anthocyanin content, while blue light enhanced the red color [32]. Thus, light signaling likely contributes to the regulation of anthocyanin biosynthesis and hence, color fading in the fruit skin of ‘Xinli 7′ pears.

We identified several plant hormone-related genes that were differentially expressed between the two pear cultivars including three *PbERF*s (LOC103960523, LOC103932979, and LOC103946144) (Supplementary Table S4). Under blue light, Py4ERF24 and Py12ERF96 were shown to interact with PyMYB114 to induce the expression of the target gene *PyUFGT*, thereby promoting anthocyanin accumulation in pears [33]. Ethylene regulates the coloration of fruits such as apples and plums [34,35,36]. Exogenous ethylene activated the expression of *MdEIL1* and *MdERF1B*—components of the ethylene signaling pathway—in apples, leading to the upregulation of *MdMYB1*, *MdMYB9*, and *MdMYB11* and an increase in anthocyanin concentration [35,36]. *PyMYB10* and *PyMYB114* expression was inhibited, and anthocyanin accumulation was blocked in pears treated with ethylene [37]. Our differential expression analysis also identified genes encoding auxin, ABA, and gibberellin. Auxin affects the coloration of red fruits such as strawberries, raspberries, and apples. Studies on apples showed that auxin promoted anthocyanin accumulation via the MdIAA121–MdARF13 module [38,39,40]. This is contrary to our results, which showed that auxin-related genes were upregulated in the fruit development stage of ‘Xinli 7′, suggesting that they are not the cause of color differences in ‘Xinqihong’ pears. Gibberellin has been shown to regulate anthocyanin levels through various signaling pathways [41]. In pears, *PbGA2ox8* induced vascular anthocyanin accumulation and contributed to the formation of red stripes on the fruit peel [42]. Consistent with these observations, gibberellin-related genes were downregulated in the fruit development stage of ‘Xinli 7′.

The RNA-seq analysis revealed 16 genes involved in calcium signaling (Supplementary Table S5). The fragments per kilobase of transcript per million mapped reads (FPKM) values of these genes were higher in ‘Xinqihong’ than in ‘Xinli 7′ at 127 DAFB. In sweet cherry, calcium foliar sprays enhanced the mechanical properties of the fruit skin and increased the levels of glucose, fructose, calcium, ascorbic acid, and anthocyanin [43]. Thus, calcium signaling may be involved in the late fading of red color in ’Xinli 7′ pear, which will be investigated in more detail in future studies.

## 4. Conclusions

**Figure 4:**
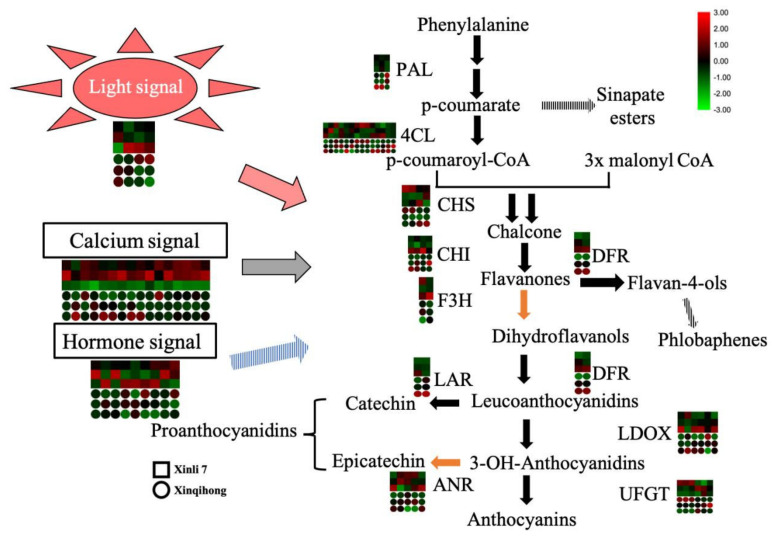
Signaling mechanisms regulating the distinct coloration patterns of ‘Xinli 7′ and ‘Xinqihong’ red pear cultivars.

The red color of red pear is determined by the pattern of anthocyanin accumulation during fruit development. In the present study, we found that the ‘Xinqihong’ variety stayed red even at maturity and fruit quality was unchanged, in contrast to ‘Xinli 7′ in which the red color faded at the late stage of fruit development. Light, calcium, and hormone signaling were found to be involved in the regulation of the color difference between the two cultivars (Figure 4). These findings provide insight into the molecular mechanisms governing color differences in red pear that may be useful for improving the quality and commercial value of the fruit.

## 5. Materials and Methods

### 5.1. Plant Materials

Samples of healthy and uniform 3-year-old ‘Xinqihong’ and ‘Xinli 7′ pear plants that had been grafted onto *P. betulifolia* Bunge rootstock were collected at Jiaodong Peninsula Regional Experimental Park (37.52° N, 120.25° E), which is in a region with a warm temperate continental monsoon climate, an average annual precipitation of 672.5 mm, and average annual temperature of 12.6 °C. For each sample, the skin of 10 fruits was removed and immediately frozen in liquid nitrogen and stored at −80 °C for total RNA isolation and measurement of pigment content.

### 5.2. Measurement of Anthocyanin, Chlorophyll, and Carotenoid Content in Peel

Approximately 1 g of fruit peel was ground to a fine powder in liquid nitrogen and extracted with 5 mL of extraction solution (1% HCl in methanol) at 4 °C for 12 h. After centrifugation at 12,000× *g* for 20 min, the supernatant was transferred to a clean tube and the absorbance at 510 nm was measured with a spectrophotometer (UV1800; Meipuda, Shanghai, China). The anthocyanin content was calculated using the equation Ca = 1000*A*V/(a*b*W), where Ca is the total anthocyanin content (mg/g), A is the absorbance value, V is the extraction solution volume, a is the absorptivity of anthocyanin (0.0775), b is the thickness of the colorimetric ware, and W is the fresh weight of fruit skin. Chlorophyll and carotenoid contents were calculated as previously described [44,45,46]. Data for 3 replicates of each sample were averaged.

### 5.3. Determination of Fruit Color

The color of apple peel was determined with a portable color difference meter (CR-400; Konica Minolta, Tokyo, Japan). The L*, a* b*, and C* values at the equator of the fruit were obtained, where L* is the brightness of the color; a* is the red–green coordinate value, which varies from −80 to 100 from green to red (with a higher absolute value indicating a deeper red or green color); b* is the blue–yellow coordinate value, which varies from −80 (blue) to 70 (yellow) (with a larger absolute value indicating a darker color); and C* is the color saturation (representing color purity).

### 5.4. RNA Extraction and First-Strand cDNA Synthesis

Total RNA was extracted and purified using the RNAprep Pure Plant Kit (Tiangen, Beijing, China) according to the manufacturer’s instructions. RNA quality was verified by spectrophotometric measurement on a NanoDrop 2000C instrument (Thermo Fisher Scientific, Waltham, MA, USA), and the integrity and purity of a 5 μL RNA sample were verified by electrophoresis on 1–1.5% agarose gel. First-strand cDNA was synthesized using the PrimeScript RT-PCR Kit (Takara, Dalian, China) according to the manufacturer’s instructions and stored at −20 °C for quantitative real-time PCR analysis.

### 5.5. Quantitative Real-Time PCR (qRT-PCR) Analysis

The cDNA templates were reverse transcribed using total RNA extracted from 5 developmental stages of the 2 red pear cultivars: Xinli 7 (87 DAFB), Xinqihong (87 DAFB), Xinli 7 (97 DAFB), Xinqihong (97 DAFB), Xinli 7 (107 DAFB), Xinqihong (107 DAFB), Xinli 7 (117DAFB), Xinqihong (117 DAFB), Xinli 7 (127 DAFB), and Xinqihong (127 DAFB). qRT-PCR amplification was carried out as follows: 95 °C for 5 min, 45 cycles at 95 °C for 15 s, 60 °C for 30 s, and 72 °C for 30 s using the Roche 480 real-time PCR system (Basil, Switzerland) at the standard mode with the FastStart Essential DNA Green Master kit. All reactions were performed in triplicate at a volume of 20 µL, containing 2 µL of 10-fold diluted cDNA, and the pear *Actin* gene was used as an internal control. Primers are listed in Supplementary Table S7.

### 5.6. Library Construction and Transcriptome Sequencing

For library construction, 1 μg of RNA per sample was used as the input material. Briefly, mRNA was purified from total RNA using poly-T oligo-coated magnetic beads. First-strand cDNA was synthesized using random hexamer primers and Moloney murine leukemia virus reverse transcriptase. Second-strand cDNA was synthesized using DNA polymerase I and RNase H. Overhangs were converted into blunt ends with exonuclease/polymerase. After adenylation of the 3′ ends of DNA fragments, an adapter with a hairpin loop structure was ligated for hybridization. cDNA fragments 370–420 bp in length were selected and PCR was performed with Phusion High-Fidelity DNA polymerase, universal PCR primers, and an index (X) primer. PCR products were purified (AMPure XP system) and library quality was assessed on the 2100 Bioanalyzer system (Agilent, Santa Clara, CA, USA). Clean reads were obtained from raw data by removing reads containing the adapter or poly-N along with low-quality reads. Q20, Q30, and the GC content of the clean data were calculated. Reference genome and gene model annotation files were downloaded from the genome website. An index of the reference genome was constructed using Hisat2 v2.0.5 (http://daehwankimlab.github.io/hisat2/, accessed on 11 February 2016), which was also used to align paired-end clean reads to the reference genome. The mapped reads of each sample were assembled with StringTie (v1.3.3b) (https://ccb.jhu.edu/software/stringtie/, accessed on 15 February 2017) using a reference-based approach. FeatureCounts v1.5.0-p3 was used to count the number of reads mapped to each gene. The FPKM of each gene was calculated based on the length of the gene and number of reads mapped to the gene. Six RNA-seq libraries were constructed for 3 developmental stages of the 2 red pear cultivars: Xinli 7_1 (87 DAFB), Xinqihong_1 (87 DAFB), Xinli 7_2 (107DAFB), and Xinqihong_2 (107 DAFB), Xinli 7_3 (127 DAFB), and Xinqihong_3 (127 DAFB). The transcriptome raw reads have been deposited at NCBI (https://www.ncbi.nlm.nih.gov/bioproject/, accessed on 18 March 2021) under accession numbers PRJNA715346.

### 5.7. Statistical Analysis

Statistical analysis was performed using Excel 2020 (Microsoft, Redmond, WA, USA). Values are represented as the mean ± SD of 3 independent biological replicates. Data were analyzed with Duncan’s test, and *p* ≤ 0.05 was considered significant.

## Figures and Tables

**Table 1:** Major intrinsic quality traits of ‘Xinqihong’ and ‘Xinli 7′ red pear varieties.

Trait	Variety	
	Xinqihong	Xinli 7
Fruit weight, g	244 ± 13.34 ^a^	209 ± 4.86 ^b^
Fruit shape	Spindle	Spindle
Fruit size, cm	11.32 × 9.99	10.5 × 8.7
Shape index	1.13 ± 0.07	1.21 ± 0.07
Color	More red	Less red
Soluble solids, %	12.4 ± 0.43 ^a^	11.2 ± 0.28 ^b^
Titratable acid, %	0.04 ± 0.01	0.06 ± 0.02
Vitamin C, mg/100 g	1.07 ± 0.04 ^b^	1.36 ± 0.04 ^a^
Fruit hardness, kg/cm^2^	5.69 ± 0.28 ^a^	4.32 ± 0.44 ^b^
Taste	Crispy	Crispy

Quantitative data are shown as the mean ± standard error (*n* = 5). Different lowercase letters are statistically significant at *p* < 0.05 (Duncan’s test).

**Table 2:** Peel color parameters of ‘Xinqihong’ and ‘Xinli 7′ red pear varieties during development.

Parameter	Variety	Days after Full Bloom				
		87	97	107	117	127
L*	Xinqihong	44.85 ± 1.21 ^c^	42.83 ± 1.38 ^c^	43.01 ± 2.89 ^c^	42.57 ± 0.80 ^c^	42.15 ± 3.32 ^c^
	Xinli 7	50.07 ± 2.62 ^bcd^	48.02 ± 4.42 ^cd^	47.42 ± 3.44 ^cd^	46.23 ± 1.95 ^d^	51.20 ± 4.02 ^bc^
a*	Xinqihong	8.84 ± 1.85 ^d^	13.28 ± 1.52 ^c^	15.13 ± 2.59 ^bc^	17.29 ± 1.99 ^b^	21.38 ± 3.24 ^a^
	Xinli 7	4.77 ± 1.02 ^b^	11.74 ± 2.39 ^a^	13.92 ± 5.41 ^a^	12.47 ± 1.57 ^a^	11.39 ± 3.48 ^a^
b*	Xinqihong	24.88 ± 2.05 ^b^	24.20 ± 1.47 ^b^	24.11 ± 3.55 ^b^	22.99 ± 1.39 ^b^	24.58 ± 3.89 ^b^
	Xinli 7	30.86 ± 1.84 ^a^	28.55 ± 3.80 ^ab^	28.80 ± 2.74 ^ab^	26.20 ± 1.51 ^b^	31.19 ± 3.34 ^a^
C*	Xinqihong	26.49 ± 1.56 ^c^	27.64 ± 1.31 ^bc^	28.68 ± 1.91 ^bc^	28.82 ± 1.43 ^bc^	32.81 ± 2.49 ^a^
	Xinli 7	31.24 ± 1.82 ^ab^	31.04 ± 2.58 ^ab^	32.39 ± 2.12 ^a^	29.06 ± 0.93 ^b^	33.41 ± 2.53 ^a^
h*	Xinqihong	70.29 ± 4.95 ^b^	61.23 ± 3.48 ^c^	57.58 ± 7.98 ^cd^	53.11 ± 3.95 ^de^	48.82 ± 7.43 ^e^
	Xinli 7	81.19 ± 1.95 ^b^	67.22 ± 7.02 ^c^	64.29 ± 10.12 ^c^	64.49 ± 3.91 ^c^	69.65 ± 6.89 ^c^

Data are shown as the mean ± standard error (*n* = 5). Different lowercase letters are statistically significant at *p* < 0.05 (Duncan’s test). L*, a*, b*, C* and h* represent fruit color different parameters.

## Supplementary Materials

The following are available online at https://www.mdpi.com/2223-7747/10/4/626/s1, Table S1: Summary of 639 DEGs in RNA-Seq, Table S2: Differentially expressed genes involved in the anthocyanin synthesis and transcription factor, Table S3: Differentially expressed genes involved in the hormone, Table S4: Differentially expressed genes involved in the calmodulin, Table S5: Differentially expressed genes involved in the Gene—Light, Table S6: Statistics of RNA-Seq data.Table S7: List of qRT-PCR Primers. Figure S1: The relative expression of the anthocyanin biosynthesis-related structural genes.
