# Supplementary material for: Differences in Anthocyanin Accumulation Patterns and Related Gene Expression in Two Varieties of Red Pear
Source: Plants (Basel). 2021 Mar 25;10(4):626. doi: 10.3390/plants10040626 (PMC8066033; doi:10.3390/plants10040626)
Supplement: Supplementary file 1 [file plants-10-00626-s001.zip › Figure S1.docx]

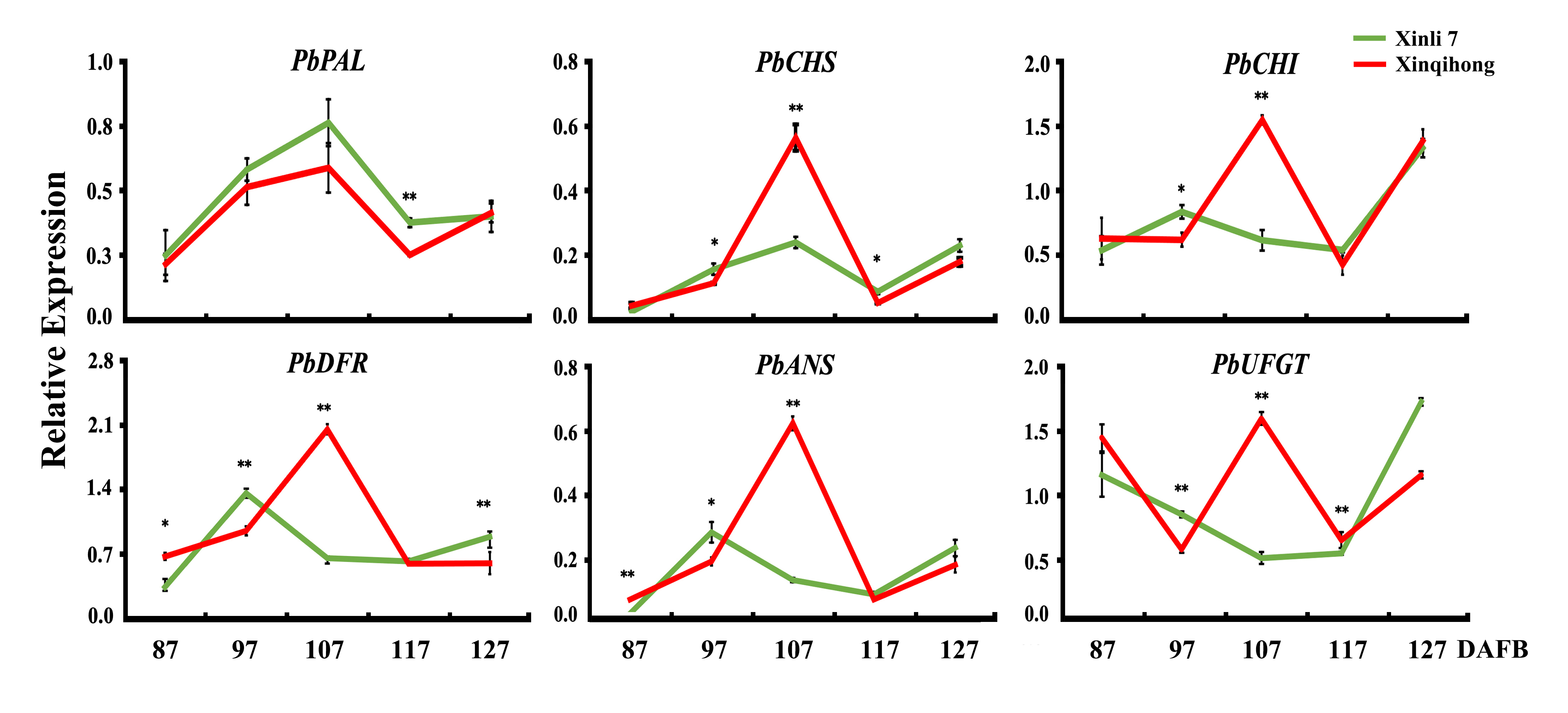


Figure S1. The relative expression of the anthocyanin biosynthesis-related structural genes. Asterisks indicate different strain using Duncan’s t test (*p < 0.05, **p < 0.01, ***p < 0.001).
